# Research trends of omics in ulcerative colitis: A bibliometric analysis

**DOI:** 10.3389/fmed.2023.1115240

**Published:** 2023-03-27

**Authors:** He Zhang, Yuanyuan Ni, Hangyu Ji, Hongliang Liu, Shaoneng Liu

**Affiliations:** ^1^Department of Gastroenterology, Guang' anmen Hospital, China Academy of Traditional Chinese Medical Sciences, Beijing, China; ^2^Office of Good Clinical Practice, Guang' anmen Hospital, China Academy of Traditional Chinese Medical Sciences, Beijing, China

**Keywords:** bibliometrics, omics, ulcerative colitis, inflammatory bowel disease, CiteSpace, VOSviewer, Bibliometrix

## Abstract

**Background:**

Omics has emerged as a promising biological science to shed light on the etiology, pathogenesis, and treatment of ulcerative colitis (UC). At present, although research on the omics of UC has drawn global attention, there is still a lack of bibliometric analysis in this field. This study aimed to access the trends and hotspots of omics in UC research.

**Method:**

Publications related to omics in UC from 1 January 2000 to 15 October 2022 were retrieved from the Web of Science Core Collection database. VOSviewer, CiteSpace, and the online bibliometric analysis platform “Bibliometrix” were adopted to extract and visualize information.

**Results:**

A total of 385 publications were finally included and the annual number of publications fluctuated. The trend in publications increased rapidly after 2019. The United States showed its dominant position in several publications, total citations, and international collaborations. The top five research organizations for publications on the research of omics in UC were Harvard Medical School, the Icahn School of Medicine at Mount Sinai, Karolinska Institutet, the Brigham and Women's Hospital, and the Massachusetts General Hospital. Ashwin Ananthakrishnan from the Massachusetts General Hospital was the most productive author, and Séverine Vermeire from the Catholic University of Leuven was co-cited most often. Inflammatory bowel disease was the most popular and co-cited journal in this field. The reference with citation bursts and trend topics showed that “ulcerative colitis,” “inflammatory bowel disease,” “microbiome,” “transcriptomics,” “genomics,” “metabolomics,” “proteomics,” “dysbiosis,” “biomarkers,” “loci,” and “therapy” are currently research hotspots.

**Conclusion:**

Our study presents several important insights into the research trends and developments in the field of omics in UC, which will provide key information for further research.

## Introduction

Omics is a rapidly evolving, comprehensive, and emerging field of study in biological science that encompasses genomics, transcriptomics, proteomics, metabolomics, and microbiomics (1). More specifically, genomics is the study of the structure, function, and inheritance of an organism's entire genome (2). Transcriptomics examines all messenger RNA molecules qualitatively or quantitatively in one cell, tissue, or organism (3). In contrast, proteomics evolved from genomics and focuses on quantifying proteins/peptides, modification, and interaction in multiple sample types by MS-based methods or high-throughput analyses (4, 5). Metabolomics is the large-scale study of multiple small molecules, such as amino acids, fatty acids, organic acids, and ketones, which are the end products of complex biochemical processes (6). More than 1,000 species of microbial cells constitute human gut microbiota, and the amount of genes present in the microbial community is 100 times greater than the human genome (7). Microbiomics, driven by the development of genomic sequencing technology, is the science of characterizing the microbial community (8). Overall, each type of omics science is typically used to identify, characterize, and quantify all biological molecules, which are associated with diseases. Omics can explore markers of disease and advance our understanding of biological pathways or processes.

Ulcerative colitis (UC) is a chronic disease where the colon and rectum become inflamed and develop ulcers or sores (9). UC occurs worldwide, with increasing incidence and prevalence (10). The prevalence rates of UC in the United States range from 214 to 286 cases per 100,000 from 2000 to 2011 (11, 12). The characteristics of UC in remission are without symptoms. However, in the period of relapsing, typical gastrointestinal presentation of UC includes bloody diarrhea, rectal urgency, mucus in the stool, and variable degrees of abdominal pain. Until now, the pathogenesis of UC is still poorly understood, which may be related to abnormal reactions of the immune system, environmental factors, and genetics (13–15).

The omics is a good way to gain insights into the etiology, pathogenesis, and treatment of UC, especially with a large number of research articles published per year. The bibliometric analysis first appeared in the late 19th and early 20th centuries (16). Nowadays, bibliometric methods are frequently adopted to decipher and map the cumulative scientific knowledge, the impact of a set of researchers, and obtain hot topics of certain research (17). The benefit of bibliometric analysis is to enable and empower scholars to gain an overview of the hotspot, co-authorship, co-citation, and the development of the specific field, which would build firm foundations for advancing a field. However, the research of omics in UC has not been assessed through bibliometric analysis. Therefore, in this study, a bibliometric analysis of publications on omics in UC was carried out. The aim of this study was to assess global trends and hotspots of omics in UC research.

## Methods

### Data source and search strategy

**Figure 1:**
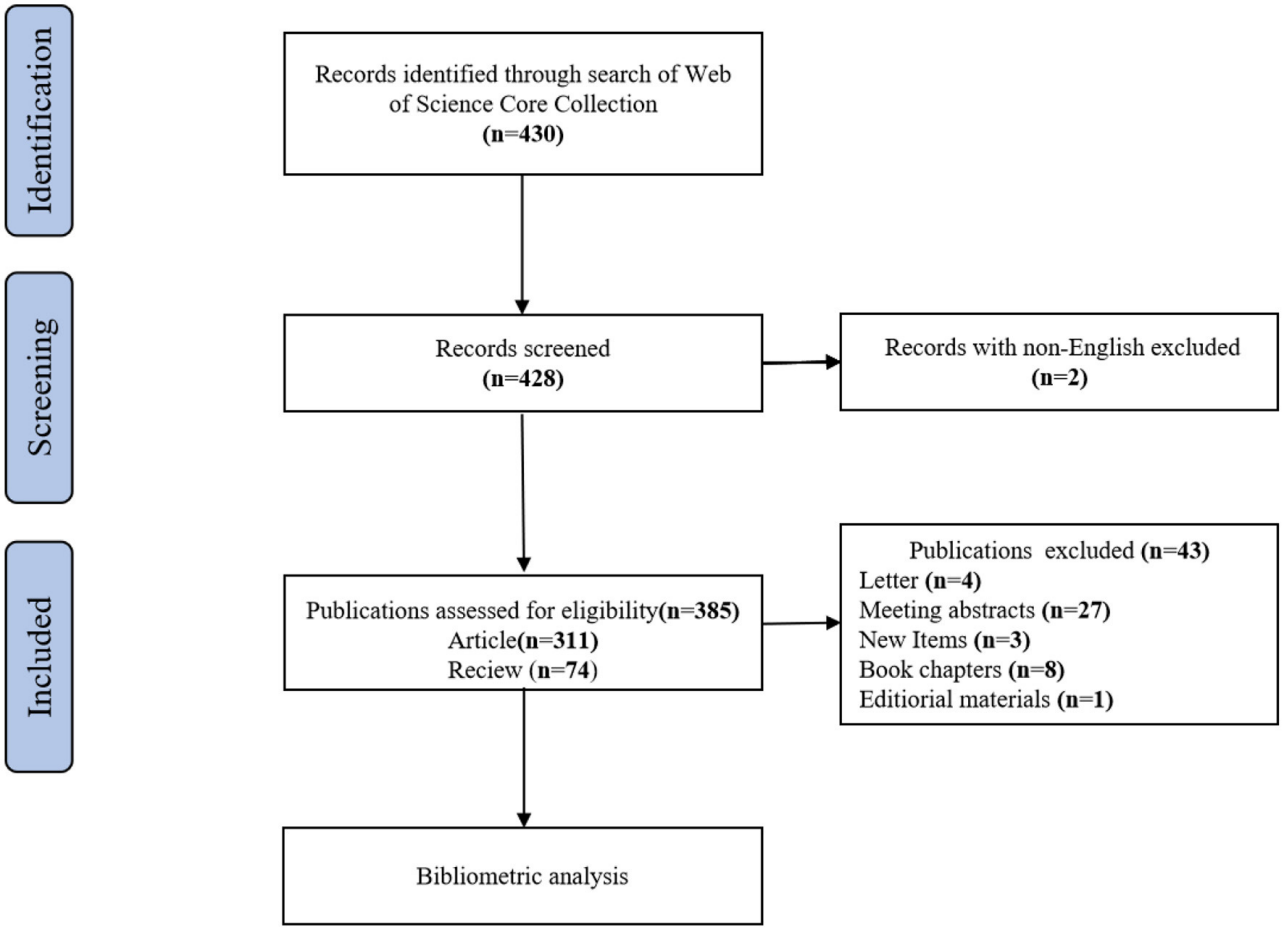
The flowchart of publications screening.

A search was conducted on the Web of Science Core Collection (WoSCC) database (https://www.webofscience.com/wos/woscc/basic-search) from 1 January 2000 to 15 October 2022 (18). The search formula was ((((((TS = (Genomics)) OR TS = (Metabolomic)) OR TS = (Transcriptomics)) OR TS = (Proteomics)) OR TS = (Microbiomics)) AND TS = (ulcerative colitis)) AND LA = (English). Only articles and review articles were included in this study. To avoid bias due to daily database updates, all searches were performed on the same day. The literature screening procedure is depicted in Figure 1.

### Data analysis

In the present study, VOSviewer (version 1.6.18), CiteSpace (version 6.1.R3), and Bibliometrix (version 3.2.1) (https://www.bibliometrix.org) were applied to analyze the data from the literature (19). The literature's authors, organizations, titles, abstracts, keywords, journals, and cited references were downloaded in plain text. VOSviewer, developed at Leiden University Centre for Science and Technology Studies, is an open-source software tool for mapping and visualizing bibliometric networks (20, 21). In our study, VOSviewer was used to analyze the co-authorship (authors, organizations, and countries), co-occurrence (author keywords), bibliographic coupling (sources), and co-citation (cited references, cited sources, and cited authors). In the networks constructed by VOSviewer, items such as author, country, organization, and keywords were represented by nodes, and the links were called edges reflecting the degree of collaboration or co-citation of each item. In order to optimize the figures display clearly, a minimum threshold was set and no more than 200 nodes were displayed in each network map. For example, 37 countries, 99 organizations, 49 most productive authors, 52 author keywords, 62 most frequently used journals, 40 co-cited references, 193 co-cited journals, and 127 co-authors were shown in the visualization maps. CiteSpace, developed by Professor Chaomei Chen of Drexel University, is a freely available application for visualizing and analyzing the literature of a scientific domain (22, 23). In this study, the dual-map overlay of journals and citation bursts was built based on CiteSpace, which helped to identify emerging trends and the distribution of academic journals in real time. The CiteSpace settings were as follows: time span (2000–2022), years per slice (1); link strength (Cosine), link scope (within slices); and selection criteria (a modified g-index in each slice). R package “Bibliometrix” was adopted to perform a global distribution of publications and the thematic evolution analysis (24). Let us install the R package and start biblioshiny digiting: library (Bibliometrix) biblioshiny. Information on scientific studies extracted from the WoSCC database. The figures were created using “biblioshiny,” a shiny application that performed a web interface for the Bibliometrix. In addition, Microsoft Office Excel 2019 was used to analyze the annual publications. The 2022 impact factor (IF) and JCR division of journals were obtained from the Web of Science group (InCites, Journal Citation Reports).

## Results

### Quantitative analysis of publication

**Figure 2:**
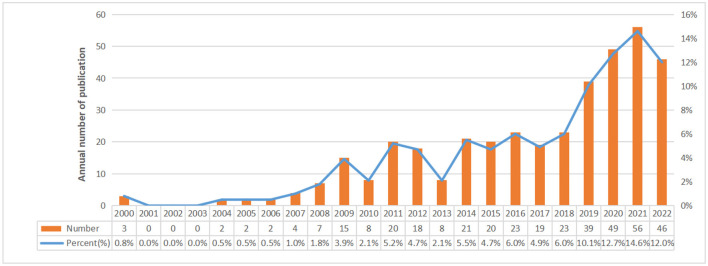
Annual number of publications in omics in UC research.

Finally, there were a total of 385 studies included, comprising 311 articles and 74 reviews on the research of omics in UC. The annual number of publications from 2000 to 2022 is shown in Figure 2. From 2000 to 2007, it was the initial stage where the annual publication number was relatively small. The number of publications from 2008 to 2018 showed continued instability, with an average annual publication number of 16.5. An outbreak of omics in UC research was witnessed from 2019 to 2022. The statistic showed the number of publications in the field of omics in UC peaked in 2021.

### Countries and institutions analysis

**Figure 3:**
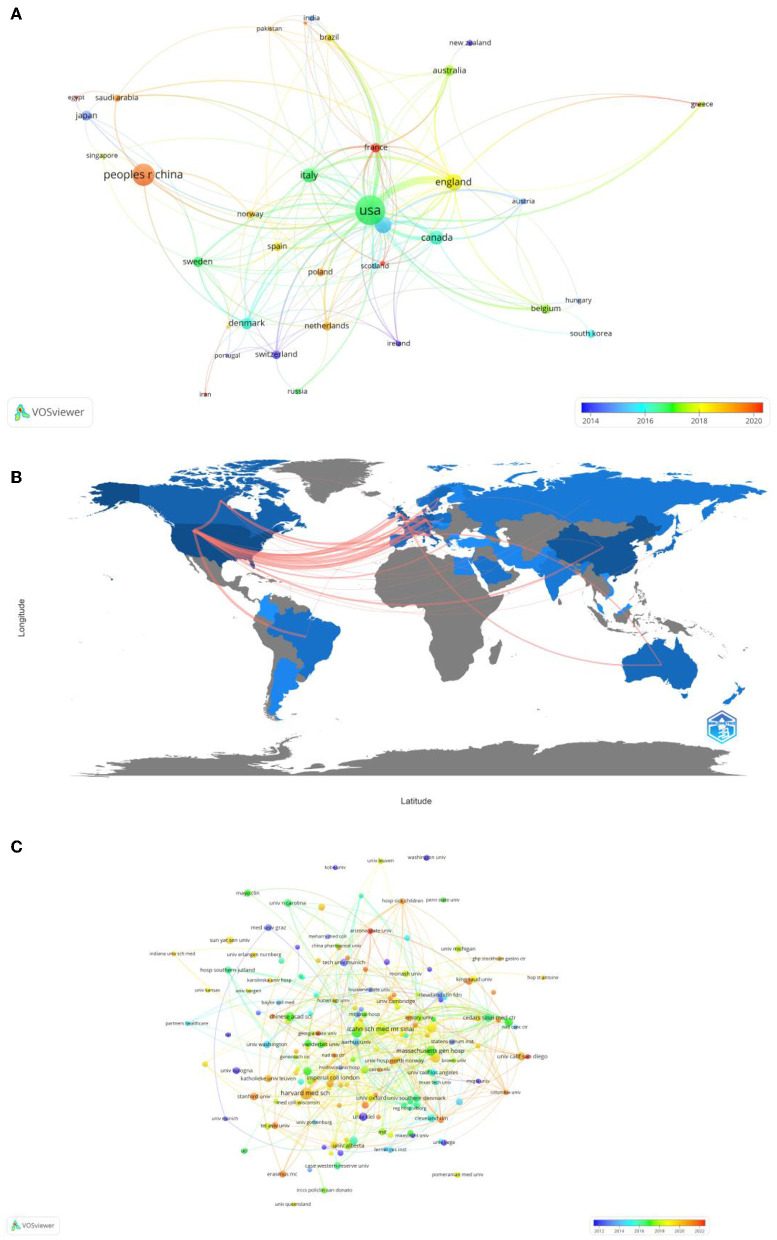
**(A)** Map of visualization of countries on research of omics in UC. **(B)** Country collaboration map. **(C)** Map of visualization of institutions on research of omics in UC.

In total, 49 countries and 809 institutions are involved in the research of omics in UC. The top 10 leading countries were distributed in North America, Asia, and Europe, as shown in Table 1. The United States (USA) (132, 22.8%) had the largest number of publications. China was in second place with 66 publications, followed by the United Kingdom (41, 7.1%) and Germany (40, 6.9%). Figure 3A presents the network of countries on the research of omics in UC, with the minimum threshold of two documents of a country. Among the 49 countries, 37 met the threshold. The network offered a clear image of seven clusters in the countries, with the highest link strength. The figure suggested the frequent coupling among the United States, United Kingdom, Germany, Italy, and Canada.

**Table 1:** The top 10 countries and institutions on the research of omics in UC.

**Rank**	**Country**	**Counts**	**Institutions**	**Counts**
1	United States	132 (22.8%)	Harvard Medical School (USA)	13 (1.6%)
2	China	66 (11.4%)	Icahn School of Medicine at Mount Sinai (USA)	12 (1.5%)
3	United Kingdom	41 (7.1%)	Karolinska Institutet (Sweden)	10 (1.2%)
4	Germany	40 (6.9%)	Brigham and Women's Hospital (USA)	9 (1.1%)
5	Canada	28 (4.8%)	Massachusetts General Hospital (USA)	9 (1.1%)
6	Italy	28 (4.8%)	Copenhagen University (Denmark)	8 (1.0%)
7	Denmark	20 (3.5%)	Broad Institute of MIT and Harvard (USA)	8 (1.0%)
8	Australia	16 (2.8%)	Chinese Academy of Sciences (China)	7 (0.9%)
9	Sweden	16 (2.8%)	Imperial College London (UK)	7 (0.9%)
10	Japan	14 (2.4%)	Harvard University (USA)	6 (0.7%)

Subsequently, a collaborative network was constructed according to the number and relationship of publications in each country (Figure 3B). The world collaboration map also highlighted collaboration and networking among countries. The blue color on the map referred to the collaboration between different countries. In addition, the pink lines between the countries reflected the degree of collaboration between the authors. The United States had the highest number of collaborations with the United Kingdom researchers (16), followed by Germany (13), Canada (8), and Brazil (7).

The 10 institutions with the largest number of publications are also presented in Table 1. Among the institutions, the top five research organizations for publications on the research of omics in UC were Harvard Medical School (13, 1.6%), the Icahn School of Medicine at Mount Sinai (12, 1.5%), Karolinska Institutet (10, 1.2%), the Brigham and Women's Hospital (9, 1.1%), and the Massachusetts General Hospital (9, 1.1%).

In Figure 3C, the institutions that met the thresholds of more than or equal to three minimum number of documents of the organization were included. The network displayed the organizations that had collaborated on the scientific documents. The size of the node referred to the total number of publications and the size of the link reflected the number of collaborations. Harvard Medical School had the highest number of scientific collaborations with international organizations, followed by the Icahn School of Medicine at Mount Sinai, the Massachusetts General Hospital, and Harvard University.

### Authors and co-cited authors

**Figure 4:**
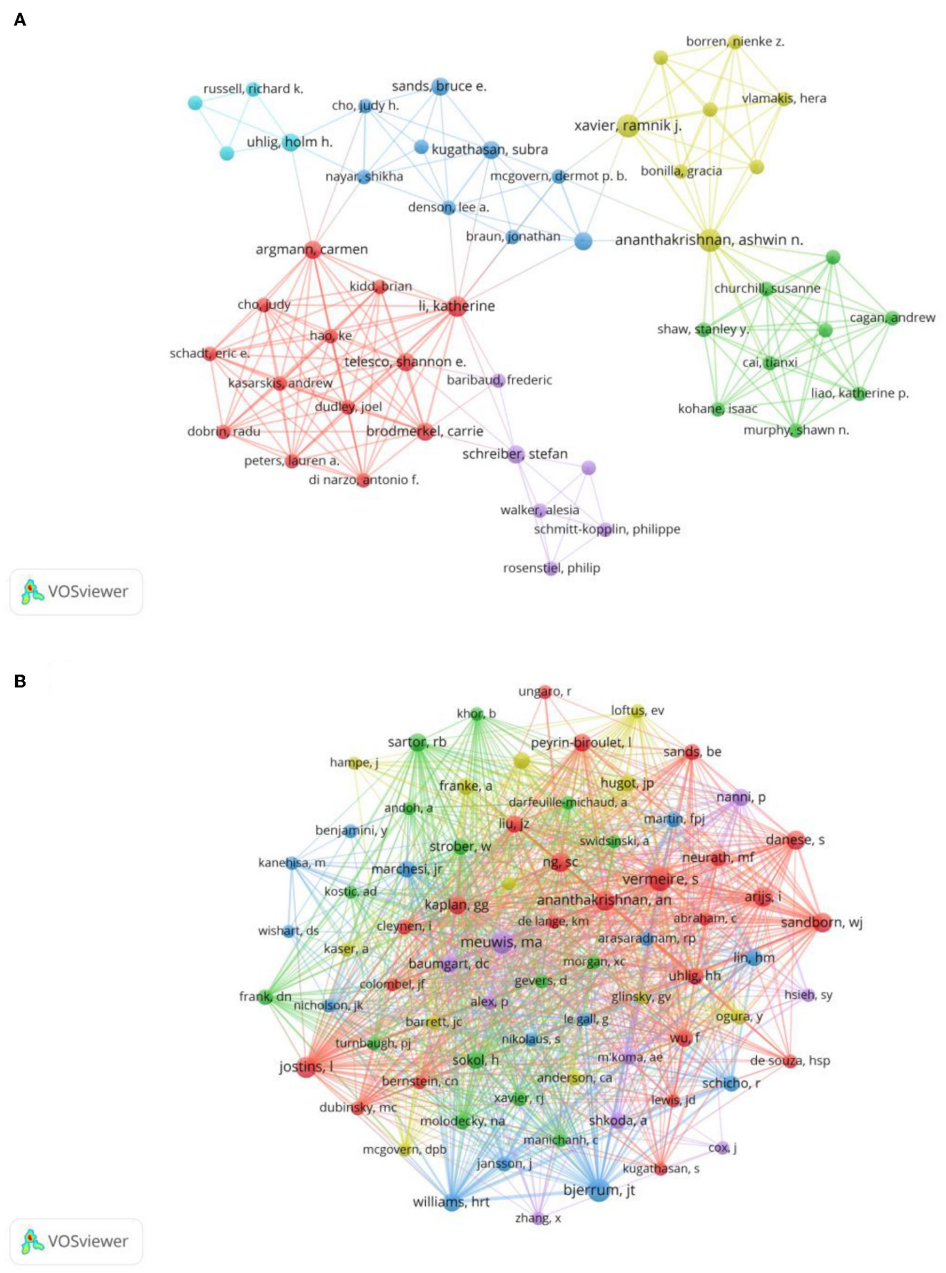
**(A)** Network of authors on research of omics in UC. **(B)** Network of co-cited authors on research of omics in UC.

A total of 2,607 authors have participated in the research of omics in UC. Detailed information of the top 10 writers based on publications is presented in Table 2. Three of them, including Ashwin Ananthakrishnan (Massachusetts General Hospital), Vibeke Andersen (University of Southern Denmark), and Ramnik Xavier (Massachusetts General Hospital) had published five articles. The author's collaborative network is presented in Figure 4A. Ashwin Ananthakrishnan, Katherine Li, Carrie Brodmerkel, Shannon E Telesco, and Carmen Argmann had the largest nodes due to the most related publications. A close collaboration existed among multiple authors. Ashwin Ananthakrishnan and Ramnik Xavier were working together. Katherine Li had close cooperation with Carrie Brodmerkel.

**Table 2:** The top 10 authors and co-cited authors on the research of omics in UC.

**Rank**	**Authors**	**Counts**	**Co-cited authors**	**Citations**
1	Ashwin N Ananthakrishnan (Massachusetts General Hospital)	5	Séverine Vermeire (Catholic University of Leuven)	66
2	Vibeke Andersen (University of Southern Denmark)	5	Jacob Tveiten Bjerrum (University of Copenhagen)	62
3	Ramnik Xavier (Massachusetts General Hospital)	5	Marie-Alice Meuwis (University of Liège)	56
4	Svend Birkelund (Aalborg Universitets forskningsportal)	4	Luke Jostins (University of Oxford)	52
5	Levinus A. Dieleman (University of Alberta)	4	Ashwin N Ananthakrishnan (Massachusetts General Hospital)	47
6	Jon Florholmen (The Arctic Universtity of Norway)	4	Siew C Ng (The Chinese University of Hong Kong)	45
7	Gennadi V Glinsky (University of California, San Diego)	4	William Jeffery Sandborn (University of California, San Diego)	45
8	Katherine/Guilin Li (Harvard University)	4	Ingrid Arijs (Hasselt University)	44
9	Karen Madsen (University of Alberta)	4	Silvio Danese (IRCCS Ospedale San Raffaele and University Vita-Salute San Raffaele)	42
10	Laurent Peyrin-Biroulet (Nancy University Hospital)	4	Gilaad G Kaplan (University of Calgary)	39

The top 10 co-authors are presented in Table 2. The most co-cited author was Séverine Vermeire (*n* = 66), followed by Jacob Tveiten Bjerrum (*n* = 62), Marie-Alice Meuwis (*n* = 56), and Luke Jostins (*n* = 52). Authors with minimum co-citations of equal to 15 were filtered to map co-author network graphs (Figure 4B). As shown in Figure 4B, the authors were presented as the nodes in the network. The size of the author node represented citation counts. There were also close collaborations among different co-cited authors, such as Séverine Vermeire, William Jeffery Sandborn, Marie-Alice Meuwis, and Jacob Tveiten Bjerrum.

### Journals and co-cited journals

**Figure 5:**
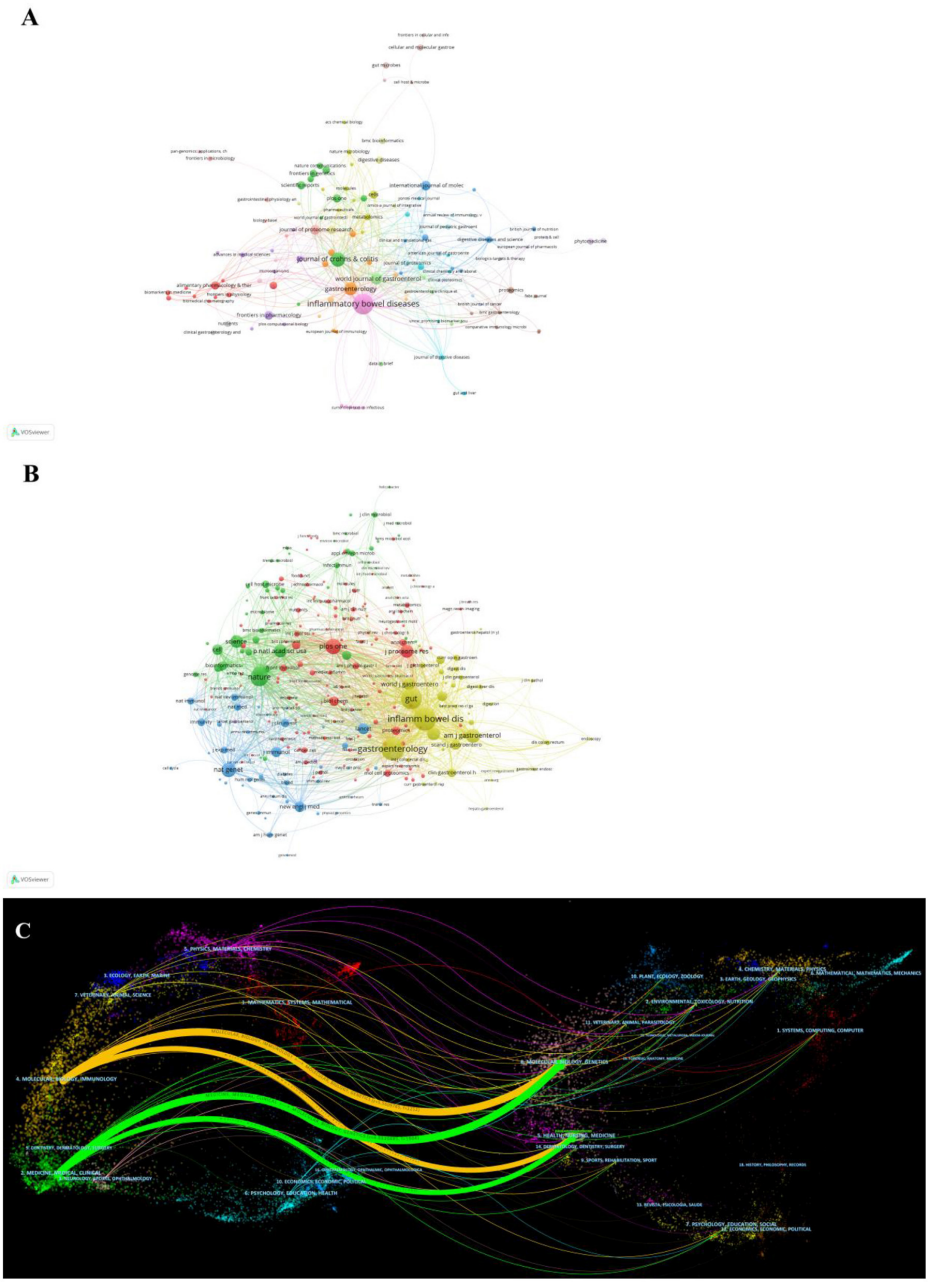
**(A)** Network of journals on research of omics in UC. **(B)** Network of co-cited journals on research of omics in UC. **(C)** The dual-map overlay of journals on research of omics in UC.

In total, publications related to omics in UC were published in 205 journals. The top 10 journals are presented in Table 3. Inflammatory bowel disease had published the most articles (*n* = 31, 15.1%), followed by Journals of Crohn's and Colitis (*n* = 12, 5.9%), Gastroenterology (*n* = 12, 5.9%), and World Journal of Gastroenterology (*n* = 10, 4.9%). The top 10 productive journals had an impact factor (IF) ranging from 3.752 to 33.883. Furthermore, the active journals appeared in the Journal Citation Reports (JCR) Q1 or Q2. The journal network is shown in Figure 5A.

**Table 3:** The top 10 journals and co-cited journals on the research of omics in UC.

**Rank**	**Journal**	**Counts**	**IF**	**JCR**	**Co-cited journal**	**Co-citation**	**IF**	**JCR**
1	Inflamm bowel dis	31 (15.1%)	7.29	Q1	Inflamm bowel dis	1223	7.29	Q1
2	J crohns colitis	15 (7.3%)	10.02	Q1	Gastroenterology	1204	33.883	Q1
3	Gastroenterology	12 (5.9%)	33.883	Q1	Gut	1005	31.795	Q1
4	World J gastroentero	10 (4.9%)	5.374	Q2	Nature	741	69.504	Q1
5	Int j mol sci	7 (3.4%)	6.208	Q1	Plos one	549	3.752	Q2
6	J proteome res	7 (3.4%)	5.37	Q1	Nat genet	497	41.376	Q1
7	Front pharmacol	6 (2.9%)	5.988	Q1	Am J gastroenterol	466	12.045	Q1
8	Sci Rep-UK	5 (2.4%)	4.997	Q2	J proteome res	396	5.37	Q1
9	Plos one	5 (2.4%)	3.752	Q2	J crohns colitis	395	10.02	Q1
10	Cells-basel	5 (2.4%)	7.666	Q2	P natl acad sci USA	390	12.779	Q1

Co-citation was called as the frequency with which two documents were cited together. The co-citation analysis of journals was considered the assessment of the influence within a particular field. As shown in Table 3, among the 2,913 co-cited journals, inflammatory bowel disease had the most co-citations (1,223), followed by Gastroenterology (1,204) and gut (1,005). Moreover, Nature (IF = 69.504) has the highest impact factor, followed by Nature Genetics (41.376). Figure 5B presents the co-citation network, which is filtered by journals' minimum co-citation equal to 20.

The dual-map overlay map is the discipline co-occurrence network, which simultaneously displays both the journals and co-cited journals. As shown in Figure 5C, the dual-map overlay of journals showed two main citation paths. The orange path, articles published in Molecular Biology/ Immunology area, and the cited publications were mostly published in journals in the fields of Molecular Biology/Genetics. The green path represented the research published in Medicine/Medical/Clinical and was mainly cited by the literature in Molecular Biology/Genetics/Health/Nurse/ Medicine.

### Co-cited references

**Figure 6:**
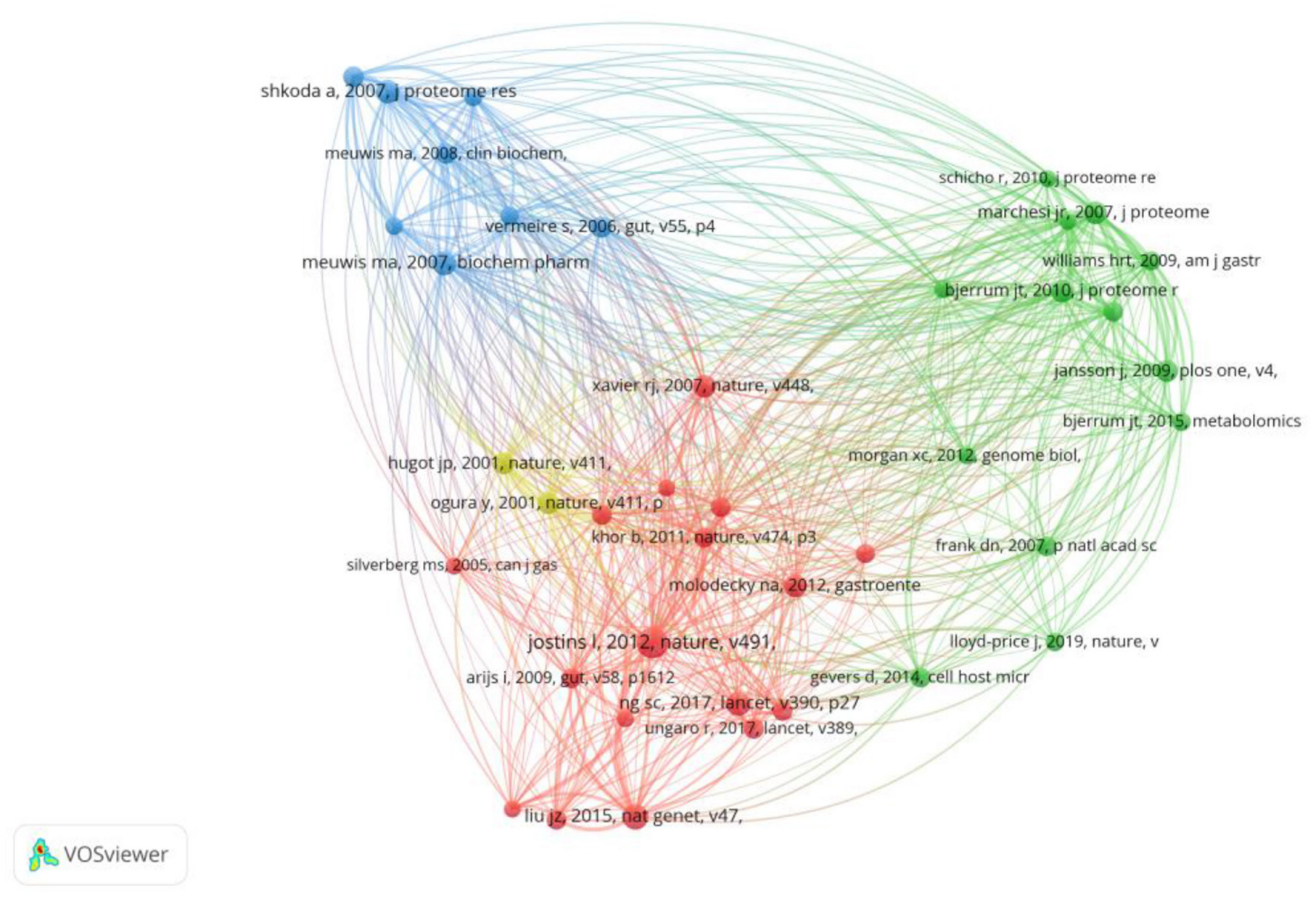
Network of co-cited references on research of omics in UC.

Co-cited references are considered as two documents cited together. From 2000 to 2022, there were 19,478 co-cited references on the topic of omics and ulcerative colitis. Of those, 26 studies had more than 20 citations. Table 4 presents the top 10 co-cited references with the most citations. *Host-microbe interactions have shaped the genetic architecture of inflammatory bowel disease* (15) published in Nature, was the most frequently co-cited (*n* = 51), followed by *association analyses identify 38 susceptibility loci for inflammatory bowel disease and highlight shared genetic risk across populations* (25), published in Nature Genetics, and *biomarker discovery for inflammatory bowel disease, using proteomic serum profiling* (26) published in Biochemical Pharmacology. The co-cited reference network map of references with a co-citation of more than or equal to 15 was constructed (Figure 6).

**Table 4:** The top 10 co-cited references on the research of omics in UC.

**Rank**	**Co-cited reference**	**Journal**	**Citations**
1	Host-microbe interactions have shaped the genetic architecture of inflammatory bowel disease	Nature	51
2	Association analyses identify 38 susceptibility loci for inflammatory bowel disease and highlight shared genetic risk across populations	Nat genet	35
3	Biomarker discovery for inflammatory bowel disease, using proteomic serum profiling	Biochem pharmacol	31
4	Worldwide incidence and prevalence of inflammatory bowel disease in the 21st century: a systematic review of population-based studies	Lancet	31
5	Differential protein expression profile in the intestinal epithelium from patients with inflammatory bowel disease	J proteome res	29
6	Metabonomics in ulcerative colitis: diagnostics, biomarker identification, and insight into the pathophysiology	J proteome res	27
7	Rapid and noninvasive metabonomic characterization of inflammatory bowel disease	J proteome res	27
8	Increasing incidence and prevalence of the inflammatory bowel diseases with time, based on systematic review	Gastroenterology	27
9	Unraveling the pathogenesis of inflammatory bowel disease	Nature	27
10	Laboratory markers in IBD: useful, magic, or unnecessary toys?	Gut	24

### Reference with citation bursts

**Figure 7:**
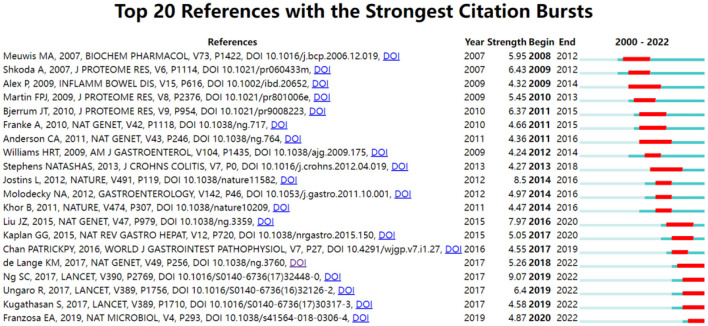
Top 20 references with the strongest citation bursts for publications on omics in UC.

Citation bursts refer to a frequency surge of publications over a period of time, which can reflect the dynamics of a certain field in part. In our study, Figure 7 shows the top 20 references with the strongest citation bursts, which are identified by CiteSpace. As shown in Figure 7, the blue line represented the time interval, and the red line depicted the year of the beginning and end of each citation burst. Citation bursts appeared as early as 2007, the strength of these 20 references ranged from 4.24 to 9.07. The reference with the strongest citation burst of 9.07 entitled “*Worldwide incidence and prevalence of inflammatory bowel disease in the 21st century: a systematic review of population-based studies*” published in The Lancet, with citation burst from 2019 to 2022, was written by Ng et al. (27). The second strongest citation burst (strength = 8.5) was entitled “*Host-microbe interactions have shaped the genetic architecture of inflammatory bowel disease*” with citation burst from 2016 to 2020, which was published in Nature (15).

### Analysis of keywords and frontiers

**Figure 8:**
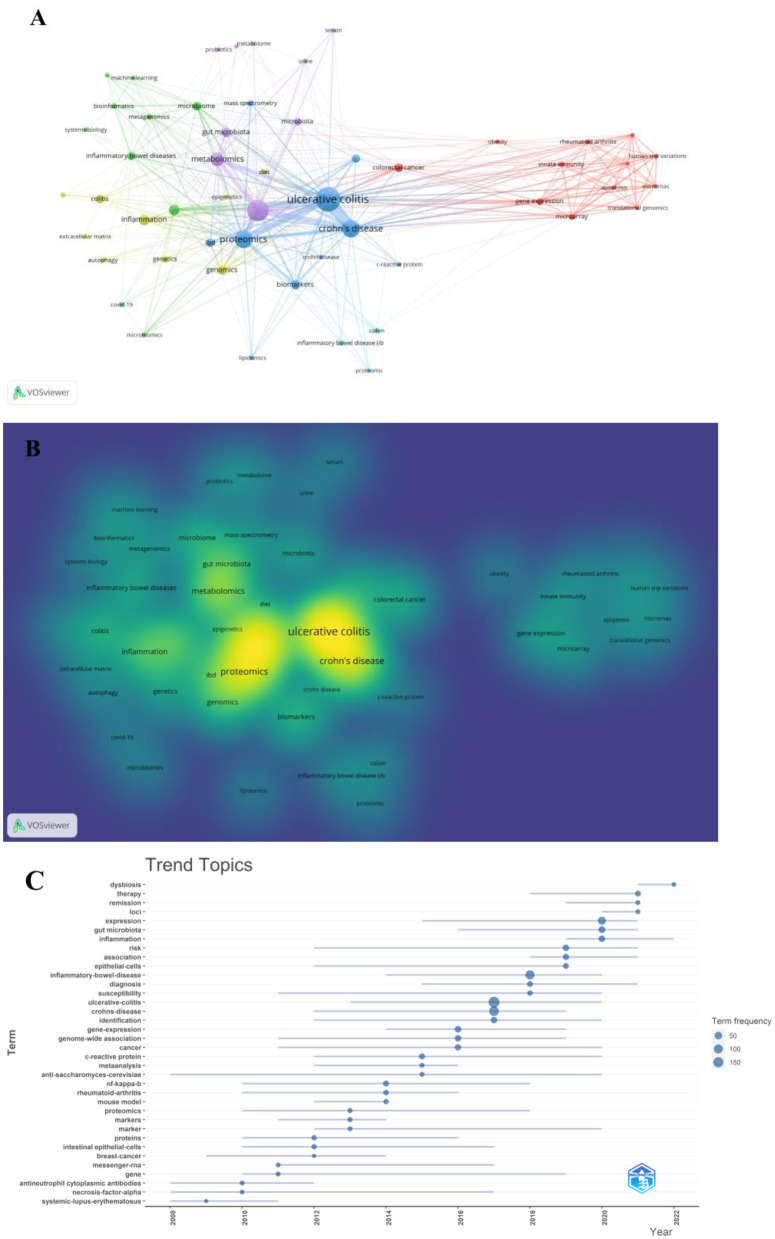
**(A)** Network of keyword on research of omics in UC. **(B)** Density map of keyword on research of omics in UC. **(C)** Trend topics.

A co-occurrence keywords map has been created based on VOSviewer to quickly capture research hotspots, as shown in Figures 8A, B. In total, 924 author keywords were found, and 53 keywords met the threshold of a minimum number of occurrences more than or equal to four. As shown in Figure 8A, six clusters were obtained in total, which was indicating six research directions. The five closest keywords in those five main clusters (red, green, blue, purple, and yellow) were as follows: (1) colorectal cancer, innate immunity, gene expression, rheumatoid arthritis, and microarray; (2) transcriptomics, inflammatory bowel disease, microbiome, bioinformatics, and systems biology; (3) ulcerative colitis, Crohn's disease, proteomics, biomarkers, and mass spectrometry; (4) inflammatory bowel disease, metabolomics, gut microbiota, microbiota, and metabolome; (5) inflammation, genomics, genetics, colitis, and extracellular matrix. Table 5 highlights the 20 most occurring keywords. In addition, the trend topic analysis of the keywords was conducted in order to gain further insights into the trends in the field of omics in UC (Figure 8C). From 2008 to 2011, the main keywords were systemic lupus erythematosus, necrosis factor alpha, and antineutrophil cytoplasmic antibodies. High-throughput sequencing has greatly assisted in research on proteomics, metabolomics, transcriptomics, genomics, and gut microbiota since 2012. Notably, these keywords of dysbiosis, gut microbiota, loci, expression, and inflammation have remarkably represented the current research hotspots of omics in UC in the past 3 years (2020–2022).

**Table 5:** The top 20 keywords on the research of omics in UC.

**Rank**	**Keywords**	**Counts**	**Rank**	**Keywords**	**Counts**
1	Ulcerative colitis	121	11	Colitis	15
2	Inflammatory bowel disease	92	12	Colorectal cancer	15
3	Proteomics	62	13	Microbiome	15
4	Crohn's disease	61	14	Biomarker	14
5	Metabolomics	38	15	Ibd	14
6	Inflammation	23	16	Inflammatory bowel diseases	13
7	Transcriptomics	23	17	Microbiota	11
8	Biomarkers	18	18	Gene expression	10
9	Genomics	18	19	Genetics	10
10	Gut microbiota	18	20	Microarray	8

## Discussion

This study adopted VOSviewer, CiteSpace, and Bibliometrix to conduct a bibliometric analysis of 385 articles from the Web of Science core database in order to identify the research hotspots and new research trends in the field of omics in UC. The results showed that the annual publications from 2000 to 2007 were extremely rare, indicating that the research foundation of omics in UC was lacking. From 2008 to 2018, the trend in publications in this field fluctuated, with an average annual publication of 16.5 articles. According to the analysis of the citation number of articles and H-index, we found the publications with high citations were located in 2012 and 2017. Therefore, during the period of 2008 to 2018, research of omics in UC was in explosive period. The number of related publications increased rapidly after 2019, indicating that research of omics in UC had entered the maturity stage of development and drew more and more researchers' attention.

The publications show a dynamic trend and change varying with years as well as the difference among the different countries. The United States, Europe, and Asia were the main regions and countries conducting research on omics in UC, especially the United States. The United States was globally dominant in publication outputs, cited authors, international collaborations, and total citations. When it came to publication numbers, the United States, China, United Kingdom, and Germany ranked favorably. Furthermore, we noticed that the United States had active cooperation with the United Kingdom, Germany, Canada, and Brazil. Although China ranked second with a large number of publications, and international collaborations and citation publications were relatively low, which may indicate that Chinese researchers should carry out extensive cooperation with foreign research institutions and promote the development of omics in UC with high-quality publications. Among the top 10 leading institutions, six institutions were located in the USA, explaining the reasons for and rapid development of omics in UC in the USA. Harvard Medical School, the Icahn School of Medicine at Mount Sinai, the Massachusetts General Hospital, and the Brigham and Women's Hospital have extensive experience in this research field. The top 10 leading institutions are quite well research platforms for collaboration and further learning.

To deeply understand the research of omics in UC, the top 10 prolific authors are summarized. Ashwin Ananthakrishnan and Ramnik Xavier are both working at the Massachusetts General Hospital. They focus on research of epidemiology and outcomes of inflammatory bowel diseases and therapy personalized medicine. Their publications of “*Multi-omics of the gut microbial ecosystem in inflammatory bowel diseases*”(28), “*Multi-omics reveal microbial determinants impacting responses to biologic therapies in inflammatory bowel disease*”(29), and “*Host-microbe interactions have shaped the genetic architecture of inflammatory bowel disease*”(15) are associated with multi-omics profiles to facilitate therapeutics for patients and serve as targets for new therapies. Vibeke Andersen, from the University of Southern Denmark, has paid much attention to differential genetic architecture and new therapeutic targets in inflammatory bowel disease (15, 30, 31). The three authors have a close collaboration. The publication with the most citations “*Host-microbe interactions have shaped the genetic architecture of inflammatory bowel disease*” was co-authored by them.

From the perspective of the co-author, Séverine Vermeireis was the most frequently cited author, followed by Jacob Tveiten Bjerrum and Jacob Tveiten Bjerrum. Séverine Vermeire coming from the Catholic University of Leuven led a research team that focused on the genetics and pharmacogenetics of inflammatory bowel disease (32). Jacob Tveiten Bjerrum is interested in the research of characterizing ulcerative colitis by metabonomics. For example, one of his publications is “*Metabonomics of human fecal extracts characterize ulcerative colitis, Crohn's disease and healthy individuals*” (33). Marie-Alice Meuwis has paid attention to proteomics studies of inflammatory bowel diseases (34, 35). Thus, the achievements of the top 10 leading co-authors laid the foundation for the research of omics in UC.

Meanwhile, most of the research on omics in UC was published in inflammatory bowel diseases (IF = 7.29, Q1), followed by Journals of Crohn's and Colitis (IF = 10.02, Q1) and Gastroenterology (IF = 33.883, Q1). The journals and co-cited journals can be divided into four groups: gastrointestinal-related journals, omics professional journals, biology and molecular journals, and comprehensive journals. Compared with journals, the citation of the journals is more high-quality international journals. From the category of journals, it demonstrates that gut, genome, and proteome are new important research areas.

### Hotspots and frontiers

**Figure 9:**
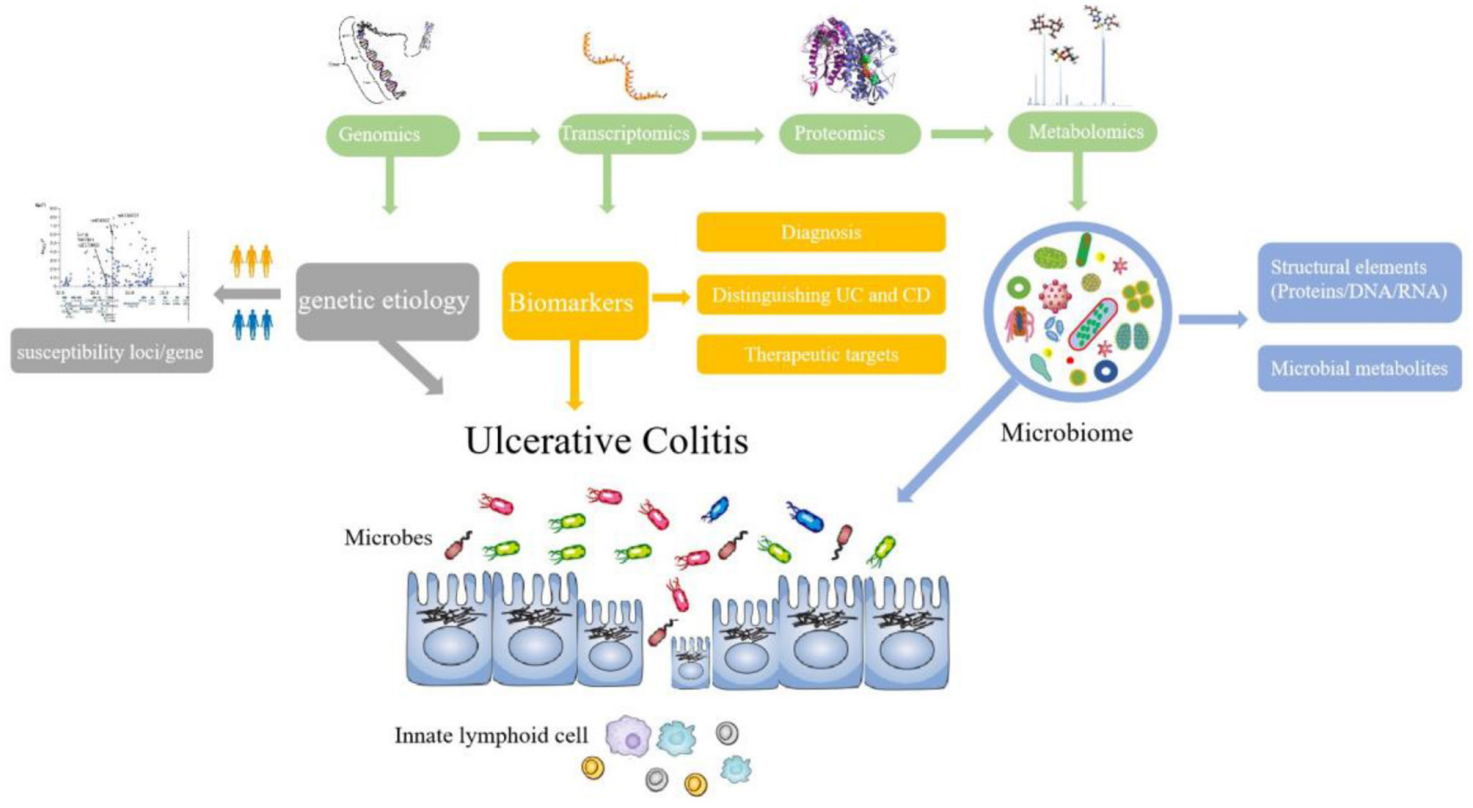
Research trend of omics in UC.

References with citation bursts and keywords represent frontiers and hotspots within a particular field (36). The reference with strong citation bursts “*Worldwide incidence and prevalence of inflammatory bowel disease in the 21st century: a systematic review of population-based studies*” pointed out that IBD was a global disease with increasing incidence and prevalence in different regions (27). The publication “*Host–microbe interactions have shaped the genetic architecture of inflammatory bowel disease*” was undertaken a meta-analysis of Crohn's disease and ulcerative colitis genome-wide association scans to emphasize the relationship and pathways shared between host mucosal immune system and microbes (15). In addition, keywords can also quickly shed light on the distribution and evolution of hotspots in the research field of omics in UC. Table 5 mainly illustrated the following keywords: ulcerative colitis, inflammatory bowel disease, proteomics, metabolomics, inflammation, transcriptomics, biomarkers, genomics, and gut microbiota. Based on keyword clusters and trend topic analysis, we summarized that the research of omics in UC mainly focused on the following aspects (Figure 9):

1. To monitor the changes in UC microbial community composition and function,

Evidence has suggested that dysbiosis of intestinal microbiota resulting in excessive intestinal inflammation contributes to the complex pathogenesis of UC (37). In patients with UC, the microbial community composition, functional diversity, and stability are compromised (38). For example, some specific bacteria associated with *Firmicutes* are decreased greatly while *Bacteroidetes* bacteria and facultative anaerobes display certain increments (39). In addition, an increase in *Desulfovibrio* and pathogenic bacteria such as *Fusobacterium varium* were also found in patients with UC (40). Omics is a good way to explore and map the intestinal microbiota change in patients with UC. Genomics, such as metagenomic analysis and microbiome genome-wide association study (mGWAS), has provided new insights into host–microbiota interactions. Several recent efforts have been made to construct genomes collection of gut microbes associated with healthy humans using metagenome sequencing, as a reference database for gut microbiota studies (41). Microbiome genome-wide association study (mGWAS) could not only identify some heritable bacterial taxa but also characterize the composition and gene contents of the intestinal microbiome associated with human health or disease (42). The interactions of intestinal microbiota and host cells occur through metabolite production, such as short-chain fatty acids (SCFAs) which have a close relationship with inflammation in the host digestive tract. SCFAs exert beneficial effects on the intestinal immune system, including regulating the recognition of intestinal epithelial cells and secretion of repair cytokine (43). Previous studies revealed that dysbiotic microbiota in patients with IBD was mainly linked to decreased abundance of SCFA species, which may affect other important metabolic pathways (44). Metabolomics focuses on the functional status of host–microbial relationships in order to identify key drivers of metabolites and metabolic pathways. The co-cited reference “*Metabonomics in Ulcerative Colitis: Diagnostics, Biomtherapyntification, And Insight into the Pathophysiology*” pointed out that patients with active UC showed antioxidants and a range of amino acids increased, while lipid, glycerophosphocholine (GPC), myo-inositol, and betaine decreased (45). In addition, the gut microbiota appears to be a promising target in the treatment of UC. Evidence was shown that stemming from a multi-omics approach, the overabundance of proteases originating from the bacterium *Bacteroides vulgatus* contributed to UC disease activity, which gain an understanding of functional microbiota alterations that drive UC and provided a strategy to treat UC (46).

2. To explore the genetic etiology of UC

Genetic susceptibility increased the chance of developing a certain disease through genetic variation. The genetic epidemiological data clearly show that genetic susceptibility to the etiology of UC is inherited. Owing to the development of genome-wide association studies (GWAS), many potential culprit genes in UC have been successfully identified, as shown in Table 6.

**Table 6:** Susceptibility to loci/gene for UC.

**Reference**	**Country**	**Sequencing cases/controls**	**Replication cases/controls**	**susceptibility loci/gene**
Fisher et al. (47)	UK	905/1,465	2,028/3,029	*IL23R, IL12B, HLA, NKX2-3* and *MST1*
Franke et al. (48)	Germany	1,167/777	1,855/3,091	*IL-10* gene on chromosome 1q32.1
Silverberg et al. (49)	North America	1,052/2,571	1,405/1,115	chromosomes 1p36 and 12q15
Asano et al. (50)	Japan	749/2,031	635/1,026	the immunoglobulin receptor gene *FCGR2A*, chromosome 13q12 and glycoprotein gene *SLC26A3*
Consortium et al. (51)	UK	2,361/5,417	2,321/4,818	chromosomes 20q13 and 16q22
Franke et al. (52)	Germany, UK, Belgium, Norway, Greece, Baltic Countries	1,043/1,703	2,539/5,428	chromosome 7q22 and at 22q13 in *IL17REL*
Julià et al. (53)	South Europe	825/1,525	1,073/1,279	chromosomes 6q22.1
Xia et al. (54)	China	382/489	764/978	*FokI* gene
Juyal et al. (55)	North India	700/761	733/1,148	*BAT2, MSH5, HSPA1L, SLC44A4, CFB* and *NOTCH4*
Wang et al. (56)	China	266/247	90/90	*RAGE* G82S
Ye et al. (57)	Korea	705/1,178	980/2,694	*IL23R, IRF5, JAK2, IL10, TNFRSF14, IL1R2, TNFSF15, YDJC, FCGR2A* and *USP12*
Saadati et al. (58)	Germany, UK	1,121/1,770	451/1,274 2,396/4,886	*KCNK9, ABCC4/MRP4* and *CLDN10*
Moon et al. (59)	Korea	24/126	793/783	rs41417449 in *BTNL2*, rs3744246 in *ORMDL3* and rs713669 in *IL17REL*

3. To diagnose and distinguish UC and Crohn's disease

Until now, the diagnosis of UC is based on clinical implications, laboratory analysis, histopathological investigation, and imaging examination. Currently, studies have demonstrated that omics can be a diagnostic tool in active and quiescent UC and also provide a differential diagnosis of Crohn's disease (CD) and UC. The most common approach to explore UC biomarkers, especially proteomics, metabonomics, and metagenomics, is to assess relative differences in proteins, metabolites, and genes between patients with UC and controls (healthy or patients with CD). Han et al. (60) compared the protein profiles of colonic mucosa in three individuals (UC, CD, and healthy controls). Twenty-seven potential biomarkers for UC, 37 biomarkers for CD and bone marrow proteoglycan (PRG2), L-plastin (LCP1), and proteasome activator subunit 1 (PSME1) for active CD were identified. In addition, UC has been associated with dramatic changes in the gut microbiota changes in the gut metabolome and gene. Thus, it is a good way to explore biomarkers associated with diagnosis and distinguishing UC through a combination of 16S rRNA gene sequencing, shotgun metagenomics, and metabolomics (61, 62). Research on candidate biomarkers for UC could not only provide insights into UC pathogenesis but also future therapeutic targets.

Furthermore, with the progress in different omics fields, it is being recognized that multi-omics variously combined with two or more omics data during analysis could provide more valuable assistance in the diagnosis, biological processes, and treatment of UC. Multi-omics profiles could demonstrate dynamic changes in gut microbiota, as well as molecular disruptions in microbial transcription, metabolite pools, and levels of antibodies in host serum during UC activity (28). Moreover, a biomarker could develop to predict disease evolution and guide stratified therapeutic approaches. Multi-omics profiles such as fecal metagenomics, serum metabolomics, and proteomics markers serve as targets for newer therapies of UC (29, 63).

Currently, there are multiple challenges in UC management due to the course of the disease and its outcome. Therefore, there is a growing need for personalized approaches to enable timely therapy and avoid a one-size-fits-all standard of treatment and care. Omics, a systems biology approach, will help to identify and validate potential biomarkers that promote personalized treatment for UC (64). With the increase of research in UC through omics technology, network biology could become a useful tool for analyzing patient data generated from various omics platforms to improve risk assessment, disease monitoring, and personalized treatment for patients with UC.

### Limitations

This study also has several limitations inherent in bibliometrics. First, a few relevant studies not included in the WoSCC database are ignored. However, WoSCC is a multidisciplinary, core journal citation index database covering approximately 34,000 journals worldwide (65). The search results can be exported from the WoSCC database and then imported into other software tools for further analysis. The WoSCC database is thought to be the most commonly used and appropriate database for bibliometric analyses. Second, only studies published in English were included, which may mean non-English publications were underestimated. Moreover, bibliometric analysis has its own weaknesses and bias. The publications with high frequency and citations may be cited for both negative as well as positive reasons. The recent publications are underrepresented due to time constraints. Nevertheless, our study still provides researchers with great objective information and insights.

## Conclusion

For this study, we used bibliometrics to analyze and evaluate the related publications on omics and UC. Generally, the number of publications in this field fluctuated. The publications increased rapidly after 2019, indicating that the research of omics in UC had attracted global attention. In total, 49 countries, 809 institutions, 2,607 authors, and 205 journals were represented in all 385 articles. The United States and China ranked favorably. The USA had active cooperation with the United Kingdom, Germany, Canada, and Brazil. Harvard Medical School topped the list of institutions with the most publications. Ashwin Ananthakrishnan from the Massachusetts General Hospital was the most productive author and Séverine Vermeire from the Catholic University of Leuven was the co-cited author most often. Moreover, inflammatory bowel disease is the most popular and co-cited journal in this field. Through the analysis of references with citation bursts and trend topics, we find “ulcerative colitis,” “inflammatory bowel disease,” “microbiome,” “transcriptomics,” “genomics,” “metabolomics,” “proteomics,” “dysbiosis,” “biomarkers,” “loci,” and “therapy” are currently research hotspots. Further studies on gut microbiota and the pathological mechanism of omics will promote understanding and targeted therapies for UC.

## Author contributions

HZ and SL design this study. HZ and YN collected and analyzed the data. HZ, HJ, and HL participated in writing the original draft. SL reviewed and revised the manuscript. All authors contributed to the article and approved the submitted version.
